# Human Milk Protein Production in Xenografts of Genetically Engineered Bovine Mammary Epithelial Stem Cells

**DOI:** 10.1371/journal.pone.0013372

**Published:** 2010-10-19

**Authors:** Eugenio Martignani, Peter Eirew, Paolo Accornero, Connie J. Eaves, Mario Baratta

**Affiliations:** 1 Department of Veterinary Morphophysiology, University of Turin, Grugliasco, Italy; 2 Terry Fox Laboratory, British Columbia Cancer Agency, Vancouver, Canada; City of Hope National Medical Center, United States of America

## Abstract

**Background:**

In the bovine species milk production is well known to correlate with mammary tissue mass. However, most advances in optimizing milk production relied on improvements of breeding and husbandry practices. A better understanding of the cells that generate bovine mammary tissue could facilitate important advances in milk production and have global economic impact. With this possibility in mind, we show that a mammary stem cell population can be functionally identified and isolated from the bovine mammary gland. We also demonstrate that this stem cell population may be a promising target for manipulating the composition of cow's milk using gene transfer.

**Methods and Findings:**

We show that the *in vitro* colony-forming cell assay for detecting normal primitive bipotent and lineage-restricted human mammary clonogenic progenitors are applicable to bovine mammary cells. Similarly, the ability of normal human mammary stem cells to regenerate functional bilayered structures in collagen gels placed under the kidney capsule of immunodeficient mice is shared by a subset of bovine mammary cells that lack aldehyde dehydrogenase activity. We also find that this activity is a distinguishing feature of luminal-restricted bovine progenitors. The regenerated structures recapitulate the organization of bovine mammary tissue, and milk could be readily detected in these structures when they were assessed by immunohistochemical analysis. Transplantation of the bovine cells transduced with a lentivirus encoding human *β-CASEIN* led to expression of the transgene and secretion of the product by their progeny regenerated *in vivo*.

**Conclusions:**

These findings point to a common developmental hierarchy shared by human and bovine mammary glands, providing strong evidence of common mechanisms regulating the maintenance and differentiation of mammary stem cells from both species. These results highlight the potential of novel engineering and transplant strategies for a variety of commercial applications including the production of modified milk components for human consumption.

## Introduction

Normal mammalian mammary tissue is a bilayered system organized into alveolar structures connected by a common ductal system. The two cell layers are separate but contiguous throughout and consist of an inner layer of cytokeratin(CK)18^+^ luminal cells and an outer layer of CK14^+^ myoepithelial cells. The luminal cells in the alveoli can produce milk, while the contractile myoepithelial cells are responsible for forcing the secreted milk to the teat cistern [1]. Studies of mammary cells from both mice and humans suggest that the luminal and myoepithelial cells are separately derived from phenotypically distinct lineage-restricted progenitors that, in turn, arise ultimately from a population of bipotent mammary cells with extensive self-renewal capacity [2]–[6]. The mammalian mammary gland is thus believed to represent a typical hierarchically organized epithelial tissue originating from a stem cell population that is maintained throughout adult life [7], [8]. Current methods for detecting human and mouse mammary progenitors require the preparation of viable single-cell suspensions and their assessment in suitable *in vitro* or *in vivo* assays to detect the growth and differentiation properties of the input cells at a clonal level. Because of the genetic homogeneity of inbred mouse strains, transplantation of cells from one mouse to another of the same strain is feasible. In addition, the use of the cleared mammary fat pad of weanling female recipient mice as an *in vivo* “niche” has proven to be an ideal system to reveal the ability of single murine mammary stem cells to regenerate a complete lactating mammary structure within 6 weeks [2], [3]. Detection of human mammary stem cells has taken advantage of the creation of genetically immunodeficient strains of mice that lack a normal complement of natural killer cells as well as B and T cells [9] to enable *in vivo* transplant assay strategy to be developed. These make use of a fibroblast-enhanced mammary fat pad or gel implants containing fibroblasts as well as the test cells to create a suitable growth-promoting environment [4], [10], [11].

To date very little information about the origin and regulation of bovine mammary cells has been generated. Ellis and Capuco [12] used a histological and morphological approach to show the existence of a small population of light staining mammary epithelial cells that constitutes approximately 50% of the proliferating mammary population. More recently Capuco [13] investigated the distribution of label-retaining epithelial cells (LREC) in pre-pubertal cows based on previous, albeit now contested [14], [15], reports that adult epithelial stem cells have this property [16], [17]. We now report the first description of bovine mammary progenitors and stem cells defined by functional growth endpoints using the same *in vitro* and *in vivo* xenotransplant systems previously developed for their human counterparts. In addition, we demonstrate the efficient transduction of these cells with a human *β-CASEIN* cDNA-encoding lentiviral vector which results in readily detectable protein production in the bovine mammary tissue subsequently regenerated in mice transplanted with transduced cells.

## Results

### Identification of bovine mammary progenitor cells

**Figure 1:**
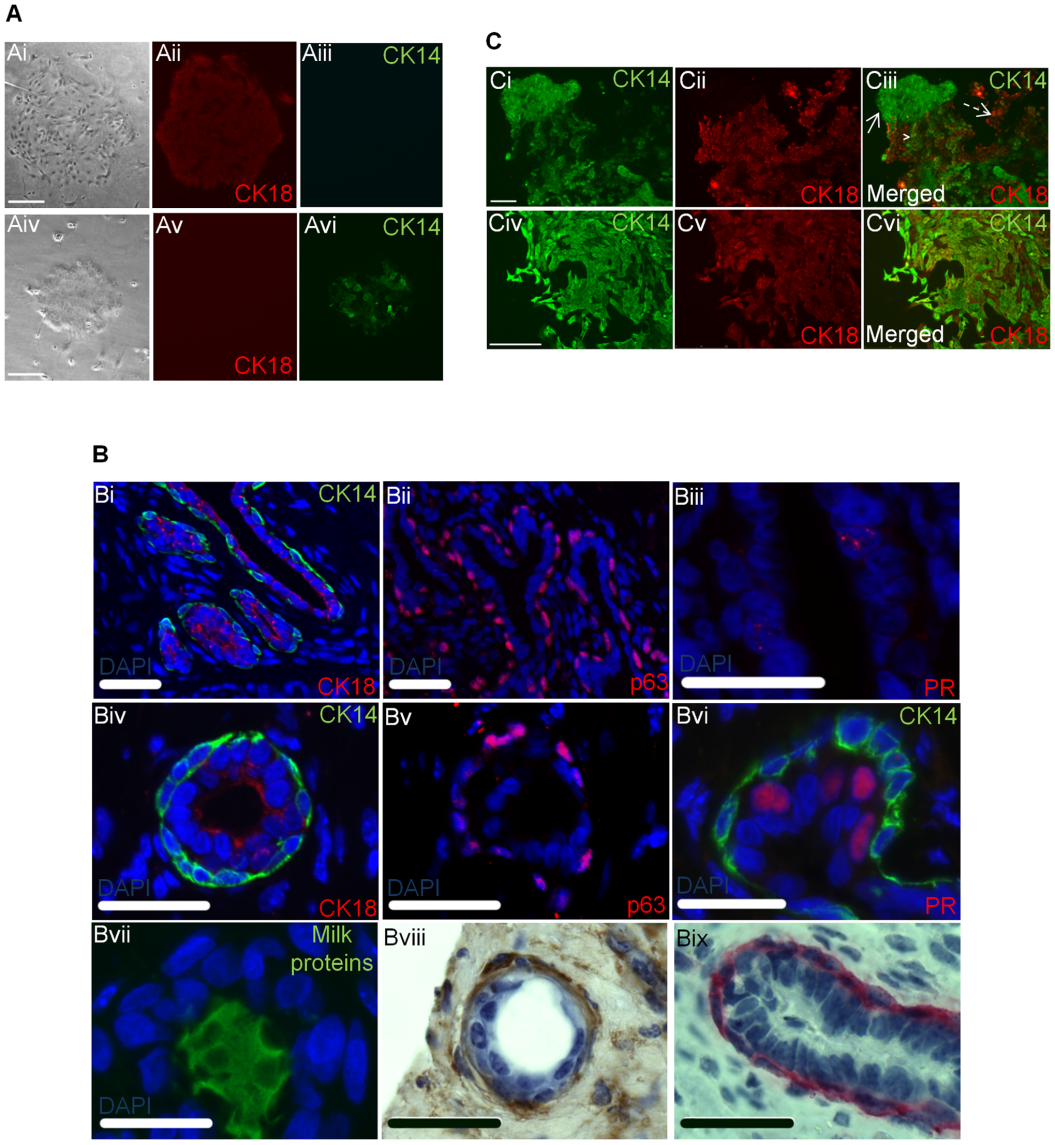
Differentiation markers expression analysis in bovine mammary colonies and xenografts. Representative pictures of a luminal-like colony (panel A, i–iii) and a myoepithelial-like colony (panel A, iv–vi) showing the differential expression of mammary lineage-restricted cytokeratins (CK). Panel B, i–iii shows the expression and spatial localization of immunofluorescently detected mammary epithelial cell markers in bovine mammary tissue. Panel B, iv–vi shows the expression and spatial localization of the same immunofluorescently detected markers in regenerated structures. The expression of milk proteins (B, vii) were detected with an Alexa-488 labelled secondary antibody, while laminin-1 (B, viii) and α-smooth muscle actin (SMA) (B, ix) were detected using DAB substrate and Fast-Red, respectively. Panel c shows representative “mixed” colonies generated in colony forming assays performed on cells from 4 weeks xenografts. Panel C, i–iii shows part of a single large colony containing CK14^+^CK18^−^ cells (arrow), CK14^−^CK18^+^ cells (dashed arrow) and CK14^+^CK18^+^ cells (arrow tip). Panel C, iv–vi shows part of another large colony where most of the cells are CK14^+^CK18^+^. Scale bars in panel A and C is 250 µm, in panel B is 25 µm.

We first sought to determine whether the methods developed for quantifying human mammary cells with *in vitro* clonogenic potential would enable the detection of analogous populations in bovine mammary tissue. Single cell suspensions were prepared from a total of eight samples of mammary tissue from cows ranging in age from 7 months to 14 years and representing different stages of mammary development (prepubertal, virgin adult and late lactation). Colony-Forming Cell (CFC) assays were then performed with these cells. In every experiment distinct large colonies were generated within 6–9 days. Colony frequencies ranged in different samples from 1 colony per 3 to 2000 cells plated. The colonies contained cells that expressed CK typical of differentiated luminal (CK18) or myoepithelial cells (CK14) (Figure 1a). Colonies containing mostly cells expressing CK14 but not CK18 were comprised of tightly packed small polygonal cells with a reduced cytoplasm. Many of these “myoepithelial” colonies contained larger cells in the center of the colony surrounded by smaller cells. Colonies containing CK18^+^ cells (“luminal” colonies) were composed of spindle-shaped cells with well defined nuclei and an extended cytoplasm. Some of these colonies contained very few cells with a very extended cytoplasm and an irregular shape. These findings are consistent with the presence in bovine mammary tissue of both luminal and myoepithelial-restricted progenitor cells each of which can proliferate *in vitro* in response to stimulation by epidermal growth factor (EGF), insulin and unknown factors produced by mouse fibroblasts.

### Bovine mammary cells regenerate mammary tissue in an in vivo xenograft assay

We next asked whether dissociated bovine mammary tissue also contained cells with *in vivo* mammary tissue regenerative activity - similar to that exhibited by mouse or human mammary stem cells. Accordingly, we transplanted additional aliquots of dissociated bovine mammary cells into cleared mammary fat pads of 3-week-old immunodeficient female NOD/SCID or NOD/SCID-IL-2 receptor gamma chain-null (NSG) mice (as per the assay for mouse mammary stem cells). Other aliquots were suspended together with mouse 10T1/2 fibroblasts in collagen gels which we then transplanted under the kidney capsule of young adult female NOD/SCID or NSG mice. Very poor growth of any mammary tissue was obtained in the cleared fat pad assay (evaluated 6 weeks later, data not shown) as previously reported for human mammary cells transplanted into this environment. In contrast, all 5 samples transplanted in the fibroblast-supplemented gels placed under the kidney capsule gave rise to histologically recognizeable mammary structures within 4 weeks (Figure 1b, iv–vi). From estimates of the number of structures seen in at least 3 groups of consecutively sectioned gels, we estimate that, on average, 1 structure was produced per 1,500 to 15,000 cells originally suspended in the gel. Most of the outgrowths appeared to be spherical in shape with a well defined lumen surrounded by 2 layers of cells. However, occasionally more complex bilayered structures with duct-like branching projections were also noted. The inner cell layer of these structures was composed of cuboidal cells that were weakly CK18^+^, consistent with a luminal phenotype. The outer layer consisted of more elongated cells with flattened nuclei and these cells were strongly positive for CK14, α-SMA and p63, consistent with a basal-myoepithelial phenotype. The regenerated structures were surrounded by laminin-1, a key component of the basement membrane that normally circumscribes the mammary gland *in vivo*. In addition, we found that the nucleus of a small number of cells within each structure stained strongly with antibodies to the progesterone receptor. Immunohistochemical evidence of milk proteins in the lumen of the pseudo-alveoli regenerated in hosts that had become pregnant was also obtained (Figure 1b, viii–ix).

To determine whether the structures produced in the gels contained detectable clonogenic progenitors, we dissociated some of the gels in collagenase to obtain single cell suspensions and then plated these *in vitro*. These experiments showed the formation of the same two types of colonies as seen in assays of freshly isolated bovine mammary cells. However, as illustrated in Figure 1c, in the assays of the dissociated regenerated structures, we also noted the presence of many “mixed” colonies (containing both CK14^+^ and CK18^+^ cells within the same colony).

### ALDH1^+^ bovine mammary cells are selectively enriched in luminal progenitors

**Figure 2:**
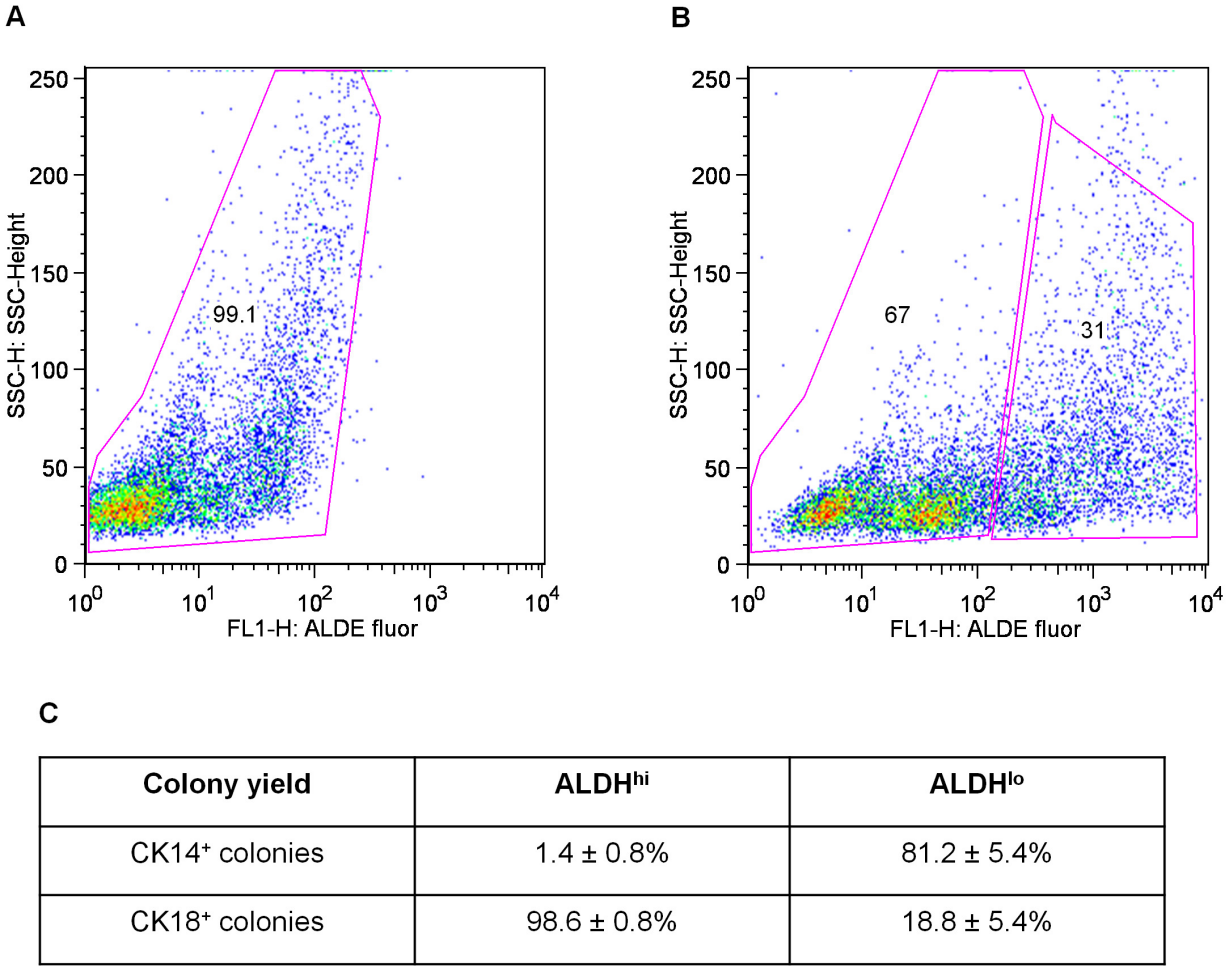
FACS profiles of bovine mammary cells stained for ALDH activity. Cells were stained with ALDEFLUOR with (a) or without (b) diethylamino-benzaldehyde (DEAB), an ALDH inhibitor. Panel c shows the proportional distribution of total clonogenic luminal and myoepithelial progenitors between the Aldehyde Dehydrogenase (ALDH)^high^ and ALDH^low^ fractions (% of all progenitors of a given type in the fraction shown ± SEM, n = 3).

We next sought to determine whether bovine cells with *in vitro* clonogenic or *in vivo* regenerative activity could be prospectively enriched within distinct subsets of cells. Unfortunately, an evaluation of antibodies against antigens expressed on primitive human and mouse mammary cells (CD24, CD29, CD49f, EpCAM and MUC1) failed to detect any that showed crossreactivity with bovine cells (data not shown). These findings precluded the adoption of most cell separation strategies previously used to discriminate subsets of primitive mouse and human mammary cells. We therefore turned to an alternative approach that allows cells to be separated based on their aldehyde dehydrogenase (ALDH) activity using a reagent that is not species-restricted. Figure 2a–b shows a representative distribution of viable cells obtained from bovine mammary tissue, in this case from a pubertal virgin cow after staining and FACS analysis of the cells according to their ALDH activity (31% ALDH^high^ and 67% ALDH1^low^). As shown in Figure 2c, assessment of the distribution of clonogenic cells in the sorted ALDH^high^ and ALDH^low^ fractions isolated from 3 different animals showed that almost all of the colonies produced by the ALDH^high^ cells had luminal features. Conversely, most of the colonies produced by the ALDH^low^ fraction had myoepithelial features (progenitor frequencies are shown in Table 1).

**Table 1:** Comparison of the frequency of clonogenic progenitors in the isolated Aldehyde Dehydrogenase (ALDH)^high^ and ALDH^low^ fractions of bovine mammary cells (n = 3).

Sample	Colony frequency	ALDH^high^ (%)	ALDH^low^ (%)
1	CK14^+^ colonies	0.13	24.00
	CK18^+^ colonies	6.38	1.63
2	CK14^+^ colonies	0.01	0.1
	CK18^+^ colonies	2.51	0.05
3	CK14^+^ colonies	0.01	0.06
	CK18^+^ colonies	0.21	0.02

Samples are: 1. 11 months old Piedmontese; 2. 7 months old Red Angus; 3. 12 months old Black Angus.

When we then assayed proportional aliquots of the same 2 fractions in the kidney capsule assay, we found that very few outgrowths were produced in the gels initially seeded with the ALDH^high^ cells (0–2 structures per gel, n = 4). In marked contrast, many extended and confluent structures were seen in the gels that had been seeded with the ALDH^low^ bovine cells (more than 15 structures per gel, n = 4). Importantly the structures obtained from the purified cells were indistinguishable histologically or by immunocytochemistry from those generated from unseparated cells.

### Expression of a human gene in bovine mammary tissues regenerated in mice from transplanted bovine mammary cells genetically engineered in vitro

**Figure 3:**
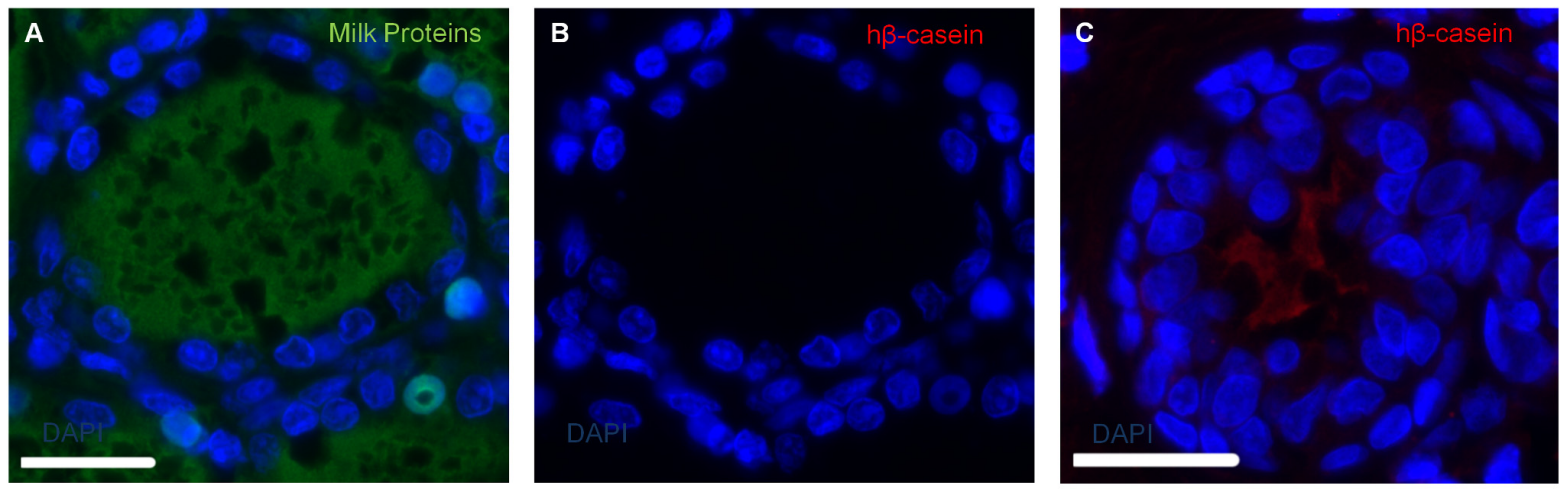
Human *β-CASEIN* expression in regenerated bovine alveoli. Representative photomicrographs showing in bovine lactating mammary tissue the presence of milk proteins in the lumen of an alveolus (a) and the absence of crossreactivity of the antibody used to detect human *β-CASEIN* (b). In (c) the expression and localization in the lumen of recombinant human *β-CASEIN* is evident in outgrowths formed by cells transduced with the human β-cas-pWPI lentiviral vector. Scale bar is 25 µm.

We created a lentiviral GFP vector encoding a human β-CASEIN cDNA and then used it to transduce sorted ALDH1^low^ mammary epithelial cells as described in the Experimental Procedures. The transduced cells were then immediately embedded in collagen gels and the gels inserted under the kidney capsule of female NOD/SCID mice which were made pregnant 1 week later. After another 3 weeks we removed the gels and subjected them to various immunohistochemical analyses. The results showed that the engineered ALDH^low^ cells had regenerated the same bilayered mammary structures as seen previously and that immunoreactive human β-CASEIN was clearly evident in the lumen of some of the regenerated structures (1 to 3 per xenograft) (Figure 3c).

## Discussion

Our findings indicate that the bovine mammary gland, like that of mice and humans, is organized as a developmental hierarchy originating in a population of phenotypically distinct mammary stem cells, each of which is endowed with the ability to regenerate and maintain a complete mammary gland structure [18]. This process appears to involve the commitment of the mammary stem cells to either a luminal (CK18^+^) or a myoepithelial (CK14^+^) differentiation pathway through a series of coordinated events that precede the ultimate loss of an ability to proliferate further. Thus under *in vitro* conditions permissive for the differentiation of both mammary cell lineages, uni-lineage colonies (containing either CK18+ or CK14+ cells but not both) may be inferred to be derived from progenitors already committed to either the luminal or myoepithelial lineages, and mixed colonies (containing both) are inferred to be derived from a more primitive uncommitted (bipotent) progenitor class. The fact that the progenitors of luminal and myoepithelial colonies can be prospectively isolated based on their ALDH activity provided strong support for this interpretation. Unexpectedly, we found that the bovine colonies containing mostly CK14^+^ cells were composed of tightly packed cells and in those containing mostly CK18^+^ cells the cells showed a looser organization. Although these morphologies are the reverse of what has been seen in similar assays of mouse and human mammary cells [5], [6], [19], the findings for bovine mammary colonies were consistent for all samples tested. Notably, the structures regenerated *in vivo* showed the same expression of markers observed in primary bovine mammary tissue with polygonal CK18^+^ cells lining the lumen surrounded by CK14^+^ cells. Given the plasticity of morphology of primary epithelial cells that can be encountered when these are cultured at low density in 2D systems, we felt more confidence in relying on CK expression to infer lineage assignments for the *in vitro* generated cells. However, this does highlight the need for caution when interpreting data from *in vitro* assays.

The observed prevalence of mixed colonies in assays of cells isolated from regenerated structures is similar to what we have seen in similar assays of human cells and likely reflects the early regenerative phase of the structures from which they were obtained. In addition, the niche requirements of these primitive bovine mammary stem cells appear to more closely resemble those of their human rather than their murine counterparts since they regenerated structures in the kidney capsule assay but not when injected directly into cleared mouse mammary fat pads [4], [10], [11].

ALDH expression has been associated with normal hematopoietic cell populations that possess a high stem/progenitor content and has also been reported to be a poor prognostic marker in several types of cancer, including breast cancer [20]–[24].

It is important to note that high ALDH activity distinguished luminal progenitors from both myoepithelial clonogenic progenitors and bovine mammary stem cells with *in vivo* regenerative activity. This adds to an extensive literature documenting a shared basal phenotype of human and murine mammary stem cells and myoepithelial progenitors and a luminal phenotype of luminal-restricted progenitors [8], [25]–[29]. This differential expression of ALDH activity allowed us to obtain an enrichment in the stem cell content of the initial suspension by selective removal of the luminal progenitors (ALDH^high^ cells). Although these ALDH results differ from those published by Ginestier C. *et al* [23] those authors used a different *in vivo* assay system and correlated their findings with cells that initiated mammosphere formation *in vitro*. Moreover, we have found that the distribution of ALDH activity amongst different types of primitive normal human mammary cells parallels that shown here for bovine mammary cells when the same types of functional assay procedures are applied (P. Eirew and C. Eaves, unpublished data).

Of particular significance in the context of this report is our demonstration of an ability of the regenerated bovine mammary structures to secrete milk proteins. The ability to introduce transgenes into ruminant mammary stem cells to produce proteins of interest, including those normally present in human milk, is an important discovery. This finding could have many applications in biotechnology considering the high milk output that can be obtained from breeds like the Holstein-Frisian and the ease of protein recovery from secreted milk. Because of the length and complexity of current procedures for obtaining transgenic cows, such a transgenic strategy in combination with the type of transplant-based approach shown here to be feasible could have significant commercial potential.

## Materials and Methods

### Mice

Female NOD/SCID mice were bred and housed at the animal facility of the Faculty of Veterinary Medicine of the University of Turin according to the procedures and guidelines approved by the Italian Ministry of Health. Female NOD/SCID-*interleukin-2 receptor-γc*-null (NSG) mice were bred and housed at the animal facility of the British Columbia Cancer Research Centre according to procedures approved by the University of British Columbia Animal Care Committee. Animal work described in this study has been reviewed and approved by the Italian Ministry of Health and the University of British Columbia Animal Care Committee, study number A06-1520. Both strains were used at 5 to 10 weeks of age as equivalent recipients for the transplants described.

### Bovine mammary tissue

Whole udders were collected from cows of different breeds (dairy and beef) and age (7 months to 14 years) at local abattoirs 30 minutes to 3 hours after slaughter. Sample collection was performed with the authorization and under the supervision of representatives of the Veterinary Services of the Italian National Health Service branch of the Ministry of Health. A piece of tissue was dissected out of the area surrounding the teats and then minced with scalpels. Approximately 10–15 grams of tissue were transferred to a 125 ml baffled Erlenmeyer flask containing 20 ml of a 1∶1 v/v mixture of Dulbecco's Modified Eagle Medium/Nutrient Mixture F12 Ham (DMEM/F12) supplemented with 2% w/v bovine serum albumin (BSA, Fraction V), 300 U/ml collagenase, 100 U/ml hyaluronidase, 100 U/ml penicillin, 100 µg/ml streptomycin (all from STEMCELL Technologies, Vancouver, BC, Canada). The tissue was then placed in a shaking incubator at 37°C for 18–20 hours. A fraction enriched in epithelial cell aggregates (organoids) was next obtained by centrifugation of the dissociated tissue at 80 g for 30 seconds and then washed in fresh DMEM/F12 medium at least 3 times. The organoids were then frozen in 6% dimethyl sulfoxide (DMSO, Fluka, Milan, Italy) containing medium and stored at −80°C until further processed. To prepare single cell suspensions, organoids were thawed and incubated with a 0.5 mg/ml trypsin solution supplemented with 0.2 mg/ml EDTA followed by vigorous pipetting for 4 minutes and subsequent washing in Hank's balanced salt solution (HBSS, STEMCELL Technologies) supplemented with 2% FBS. Cells were then treated with 5 mg/ml dispase and 100 µg/ml DNAseI (STEMCELL Technologies) and passed through a 40 µm cell strainer (BD Biosciences, San Jose, CA, USA) to remove remaining cell aggregates.

### Colony-Forming Cell (CFC) assay

60 mm tissue culture dishes were coated with collagen by incubation for 1 hour at 37°C with a solution of rat tail type 1 collagen (80 µl of rat tail type I collagen @ 3.66 mg/ml, BD Bioscience diluted in 50 ml of a 2% acetic acid solution (STEMCELL Technologies). Bovine mammary cells were then added together with 2×10^5^ NIH 3T3 mouse fibroblasts previously either irradiated with 50 Gy 250 KvP X-rays or treated with 10 µg/ml mitomycin C (Sigma-Aldrich, Milan, Italy) for 2 hours in human EpiCult B medium supplemented with 5% FBS, 10^−6^ M hydrocortisone (Sigma-Aldrich), 100 U/ml penicillin and 100 µg/ml streptomycin. The assay dishes were then incubated at 37°C with 5% CO_2_ for 24 hours at the end of which the medium was exchanged for fresh medium of the same composition but without FBS. Cells were incubated for another 6–9 days and then the cultures were fixed with acetone/methanol (1∶1 v/v, Fluka) and either stained with Wright's Giemsa (Sigma-Aldrich) or with a crystal violet solution (50 mg crystal violet in a 20% methanol solution, Sigma-Aldrich). Fixed colonies were immunostained (as described below) with antibodies to human CK14 (1∶500 dilution, polyclonal AF-64, Covance, Princeton, NJ, USA), CK18 (1∶200 dilution, clone KS-B17.2, Sigma-Aldrich) and α-smooth muscle actin (α-SMA, 1∶100 dilution, clone 1A4, Sigma-Aldrich), after validation that all of these cross-reacted with bovine antigens. Colonies containing more than 50 cells (after 7 days of culture) or than 100 cells (after 10 days of culture) were then counted and progenitor frequencies expressed as the total number of colonies obtained per 100 cells seeded.

### Xenotransplants

Concentrated rat tail collagen was prepared as previously described [30]. A ready to use collagen solution was made by mixing 88% concentrated collagen, 10% 10× DMEM and 2% 10 M NaOH to neutralize the pH and kept on ice. Collagen gels were prepared as previously described [4]. Each gel contained 1.6×10^5^ 10T1/2 fibroblasts previously irradiated with 15 Gy 250 KvP X-rays or treated with 2 µg/ml mitomycin C and 2.5×10^4^–7.5×10^4^ bovine primary mammary cells. The gels were kept on ice until surgically implanted.

Anesthetized mice were shaved in the posterior dorsal area and cleaned with 70% ethanol. At first a 2 cm anterior-to-posterior cut was made through the skin along a median line followed by a smaller incision of approximately 4–5 mm in the abdominal wall directly above the kidney position. The kidney was then gently pushed out through the cut, the kidney capsule was lifted and a short incision of 2–3 mm was made in it. The collagen gels were then pushed under the capsule using fire polished glass pipette tips. The abdominal wall was then sutured and the procedure was repeated on the contra-lateral kidney. In some experiments a slow-release pellet containing 2 mg β-estradiol (Sigma-Aldrich) and 4 mg progesterone (Sigma-Aldrich) in silicone (MED-4011, NuSil Technology, Carpinteria, CA, USA) was placed subcutaneously before finally suturing together the skin of the mouse. In other experiments the mice were mated 1 or 3 weeks after the surgery.

### Cell separation

Bovine mammary cells to be stained with labeled antibodies were first incubated in HBSS supplemented with 2% FBS. Antibodies evaluated were fluorescein isothiocyanate (FITC)-conjugated mouse antibody to human EpCAM (clone VU1-D9, STEMCELL Technologies), allophycocyanin (APC)-conjugated rat antibody to human CD49f (clone GOH3, R&D systems, Minneapolis, MN, USA), phycoerythrin(PE)-conjugated rat antibody to mouse CD24 (clone M1/69, BD Biosciences Pharmingen,San Diego, CA, USA), APC-Cy7-conjugated mouse antibody to human CD29 (clone TS2/19, Biolegend, San Diego, CA, USA), unconjugated mouse antibody against human MUC1 (clone 214D4, STEMCELL Technologies) and unconjugated mouse antibody against human CD24 (clone 8.B.76, Abcam, Cambridge, MA, USA). When unconjugated purified antibodies were used, incubation with the primary antibodies was followed by labeling with a PE-conjugated goat antibody against mouse IgG (Jackson ImmunoResearch, West Grove, PA, USA). Cells were subsequently analyzed with a FACSCalibur (Becton Dickinson, San Jose, CA, USA).

Bovine mammary cells with ALDH activity (ALDH^high^ cells) were detected after staining using the ALDEFLUOR Assay Kit (STEMCELL Technologies) according to the supplier's instructions and a 1 µg/ml propidium iodide (PI, Sigma-Aldrich) solution to discriminate live cells. ALDH^high^ and ALDH^low^ cells were isolated using a FACSVantage SE (Beckton Dickinson).

### Immunostaining

Four to 7 µm sections were dewaxed and processed either for immunohistochemistry or immunofluorescence. Briefly, after dewaxing, sections were placed in Tris-HCl buffered saline supplemented with Tween-20 (TBS-Tween, 0.1 M Tris HCl, 0.14 M NaCl, 0.05% Tween-20, pH 7.6, all reagents from Sigma-Aldrich) for 10 minutes, then incubated at room temperature for 1 hour in TBS-Tween supplemented with 10% goat serum (Sigma-Aldrich) and then for 1 hour at room temperature with one or two primary antibodies as required. Sections were then washed 3 times with TBS-Tween, secondary antibodies applied, and sections incubated for another hour. Where alkaline phosphatase (AP) conjugated polymer (Envision kit, DAKO, Glostrup, Denmark) or horseradish peroxidase (HRP)-conjugated secondary antibody (Discovery Universal Secondary Antibody kit, Ventana, Tucson, AZ, USA) was to be used, sections were treated with a suitable substrate (BCIP®/NBT from Sigma or Fast-Red from Thermo Scientific for AP, 3,3′-diaminobenzidine, DAB, from Ventana for HRP). Sections were then counterstained with either Meyer's hematoxylin (Bio-Optica, Milan, Italy) or 4′,6-diamidino-2-phenylindole (DAPI, Sigma-Aldrich) and then mounted. To stain colonies grown in tissue culture treated dishes, cells were fixed with methanol/acetone (1∶1 v/v) for 30 seconds to 1 minute, blocked with TBS-Tween supplemented with 10% goat serum and then stained as described above.

Primary antibodies used were antibodies to human CK14, CK18, α-SMA, p63 (1∶200 dilution, clone 4A4, Thermo Fisher Scientific, Fremont, CA, USA), Laminin-1 (1∶50 dilution, polyclonal, Sigma-Aldrich), progesterone receptor (PR, 1∶70 dilution, clone hPRa 2, Thermo Fisher Scientific), milk proteins (1∶500 dilution, polyclonal Nordic Immunology, Tilburg, Netherlands), and β-casein (1∶70 dilution, clone F14.20, Harlan Laboratories, Madison, WI, USA). Secondary antibodies used were AlexaFluor® 488-labelled goat anti-rabbit IgG and AlexaFluor® 594-labelled goat anti-mouse IgG (both from Invitrogen, Carlsbad, CA, USA).

Negatively stained controls were performed for each antigen by replacing the primary antibody with a suitable isotype (normal mouse IgG or normal rabbit IgG from Santa Cruz Biotechnology Inc., Santa Cruz, CA, USA) at the same concentration.

### Generation of a human β-casein lentiviral vector

The NIH Mammalian Gene Collection was searched for a full human β-CASEIN cDNA clone (Acc. No. BC096197). A pCR-*BluntII*-TOPO vector containing the full cDNA (IRAMp995F082Q) was provided by ImaGenes (Berlin, Germany). The β-CASEIN open reading frame was cut from the backbone using *Eco*RI (Fermentas, St. Leon-Rot, Germany) digestion and ends blunted by incubation with T4 polymerase (Roche Applied Science, Monza, Italy). The fragment was then purified and cloned into the *Pme*I site of the pWPI plasmid (Addgene, Cambridge, MA, USA) to generate pWPI-β-cas. The insert orientation was tested by size analysis of *Kpn*I/*Nde*I double digestion fragments. A VSVG enveloped pWPI-β-cas lentiviral vector (Lenti-β-cas-GFP) preparation was produced by transfecting plasmids from a 3^rd^ generation system (pMDLg/pRRE, pRSV-Rev, pMD2.G, Addgene) into 293 T cells and the preparation was then concentrated by ultracentrifugation (25,000 rpm for 90 minutes at 4°C), aliquoted, titrated and frozen in liquid nitrogen. HeLa cells were incubated with the Lenti-β-cas-GFP vector, infection confirmed by detection of GFP and human β-CASEIN transgene expression measured in extracted RNA by Real Time (RT)-PCR. For this RNA was extracted using the RNeasy Mini Kit (QIAGEN, Mississauga, ON, Canada), reverse-transcribed with SuperScript II Reverse Transcriptase (Invitrogen) following the manufacturer's protocols and then RT-PCR performed.

### Infection of bovine primary mammary cells

5×10^5^–1×10^6^ isolated bovine mammary cells were resuspended in 120 µl HBSS supplemented with 2% FBS and Lenti-β-cas-GFP added at a multiplicity of infection (m.o.i.) of 20. The volume was then adjusted to 200 µl with HBSS and the cells incubated for 4 hours on ice. After washing the cells, half of them were seeded into a tissue culture dish to evaluate the infection efficiency (% GFP+ cells after 24–48 hours in vitro) and the remaining cells were used immediately for kidney xenografts.

### Real Time RT-PCR

The quantity of human β*-CASEIN* transcript expression was measured relative to the amount of bovine *Glyceraldehyde 3-phosphate dehydrogenase* (*GAPDH*) that was used as housekeeping gene to correct for RNA concentration and reverse transcription efficiency. PCR amplification was carried out using iQ SYBR Green Supermix (Bio-Rad, Segrate, Italy) in a MJ Mini Cycler equipped with a MJ Mini-Opticon Real Time PCR detector. Primers for human β*-CASEIN* were: forward 5′-GCA GCA AGG AGA GGA TGA-3′; reverse 5′-GGT ATC GTT GGA GAT TTA AG-3′. Primers for *GAPDH* were: forward 5′-TGCACCACCAACTGCTTAGC-3′; reverse 5′-GGCATGGACTGTGGTCATGAG-3′. 45 PCR cycles were set as following: cycle 1, 94°C for 2′; cycle 2, 94°C for 45″, 45 cycles at 55°C for 30″. Quantification was done using the Δ (ΔC_t_) method as described by Livak and Schmittgen[31].

## References

[1] Capuco AV, Ellis S, Wood DL, Akers RM, Garrett W (2002). Postnatal mammary ductal growth: three-dimensional imaging of cell proliferation, effects of estrogen treatment, and expression of steroid receptors in prepubertal calves.. Tissue Cell.

[2] Stingl J, Eirew P, Ricketson I, Shackleton M, Vaillant F (2006). Purification and unique properties of mammary epithelial stem cells.. Nature.

[3] Shackleton M, Vaillant F, Simpson KJ, Stingl J, Smyth GK (2006). Generation of a functional mammary gland from a single stem cell.. Nature.

[4] Eirew P, Stingl J, Raouf A, Turashvili G, Aparicio S (2008). A method for quantifying normal human mammary epithelial stem cells with in vivo regenerative ability.. Nat Med.

[5] Stingl J, Raouf A, Emerman JT, Eaves CJ (2005). Epithelial progenitors in the normal human mammary gland.. J Mammary Gland Biol Neoplasia.

[6] Raouf A, Zhao Y, To K, Stingl J, Delaney A (2008). Transcriptome analysis of the normal human mammary cell commitment and differentiation process.. Cell Stem Cell.

[7] Smith GH, Medina D (1988). A morphologically distinct candidate for an epithelial stem cell in mouse mammary gland.. J Cell Sci.

[8] Kordon EC, Smith GH (1998). An entire functional mammary gland may comprise the progeny from a single cell.. Development.

[9] Shultz LD, Schweitzer PA, Christianson SW, Gott B, Schweitzer IB (1995). Multiple defects in innate and adaptive immunologic function in NOD/LtSz-scid mice.. J Immunol.

[10] Kuperwasser C, Chavarria T, Wu M, Magrane G, Gray JW (2004). Reconstruction of functionally normal and malignant human breast tissues in mice.. Proc Natl Acad Sci U S A.

[11] Lim E, Vaillant F, Wu D, Forrest NC, Pal B (2009). Aberrant luminal progenitors as the candidate target population for basal tumor development in BRCA1 mutation carriers.. Nat Med.

[12] Ellis S, Capuco AV (2002). Cell proliferation in bovine mammary epithelium: identification of the primary proliferative cell population.. Tissue Cell.

[13] Capuco AV (2007). Identification of putative bovine mammary epithelial stem cells by their retention of labeled DNA strands.. Exp Biol Med (Maywood).

[14] Kiel MJ, He S, Ashkenazi R, Gentry SN, Teta M (2007). Haematopoietic stem cells do not asymmetrically segregate chromosomes or retain BrdU.. Nature.

[15] Barker N, van Es JH, Kuipers J, Kujala P, van den Born M (2007). Identification of stem cells in small intestine and colon by marker gene Lgr5.. Nature.

[16] Shinin V, Gayraud-Morel B, Gomes D, Tajbakhsh S (2006). Asymmetric division and cosegregation of template DNA strands in adult muscle satellite cells.. Nat Cell Biol.

[17] Smith GH (2005). Label-retaining epithelial cells in mouse mammary gland divide asymmetrically and retain their template DNA strands.. Development.

[18] Stingl J, Raouf A, Eirew P, Eaves CJ (2006). Deciphering the mammary epithelial cell hierarchy.. Cell Cycle.

[19] Asselin-Labat ML, Sutherland KD, Barker H, Thomas R, Shackleton M (2007). Gata-3 is an essential regulator of mammary-gland morphogenesis and luminal-cell differentiation.. Nat Cell Biol.

[20] Huang EH, Hynes MJ, Zhang T, Ginestier C, Dontu G (2009). Aldehyde dehydrogenase 1 is a marker for normal and malignant human colonic stem cells (SC) and tracks SC overpopulation during colon tumorigenesis.. Cancer Res.

[21] Burger PE, Gupta R, Xiong X, Ontiveros CS, Salm SN (2009). High aldehyde dehydrogenase activity: a novel functional marker of murine prostate stem/progenitor cells.. Stem Cells.

[22] Jean E, Laoudj-Chenivesse D, Notarnicola C, Rouger K, Serratrice N (2009). Aldehyde dehydrogenase activity promotes survival of human muscle precursor cells.. J Cell Mol Med.

[23] Ginestier C, Hur MH, Charafe-Jauffret E, Monville F, Dutcher J (2007). ALDH1 is a marker of normal and malignant human mammary stem cells and a predictor of poor clinical outcome.. Cell Stem Cell.

[24] Storms RW, Green PD, Safford KM, Niedzwiecki D, Cogle CR (2005). Distinct hematopoietic progenitor compartments are delineated by the expression of aldehyde dehydrogenase and CD34.. Blood.

[25] Stingl J (2009). Detection and analysis of mammary gland stem cells.. J Pathol.

[26] Badders NM, Goel S, Clark RJ, Klos KS, Kim S (2009). The Wnt receptor, Lrp5, is expressed by mouse mammary stem cells and is required to maintain the basal lineage.. PLoS One.

[27] Hanker L, Karn T, Ruckhaeberle E, Gaetje R, Solbach C (2010). Clinical relevance of the putative stem cell marker p63 in breast cancer.. Breast Cancer Res Treat.

[28] Tiede BJ, Owens LA, Li F, DeCoste C, Kang Y (2009). A novel mouse model for non-invasive single marker tracking of mammary stem cells in vivo reveals stem cell dynamics throughout pregnancy.. PLoS One.

[29] Brisken C, Duss S (2007). Stem cells and the stem cell niche in the breast: an integrated hormonal and developmental perspective.. Stem Cell Rev.

[30] Richards J, Pasco D, Yang J, Guzman R, Nandi S (1983). Comparison of the growth of normal and neoplastic mouse mammary cells on plastic, on collagen gels and in collagen gels.. Exp Cell Res.

[31] Livak KJ, Schmittgen TD (2001). Analysis of relative gene expression data using real-time quantitative PCR and the 2(-Delta Delta C(T)) Method.. Methods.

